# Supplementary material for: Prophage-mediated lysogenic conversion drives virulence evolution and genomic plasticity in Streptococcus suis serotype 9
Source: Microbiol Spectr. 2026 Apr 13;14(5):e00061-26. doi: 10.1128/spectrum.00061-26 (PMC13141850; doi:10.1128/spectrum.00061-26)
Supplement: Supplemental material — Fig. S1 to S3; Tables S1 and S2. [file spectrum.00061-26-s0001.docx]

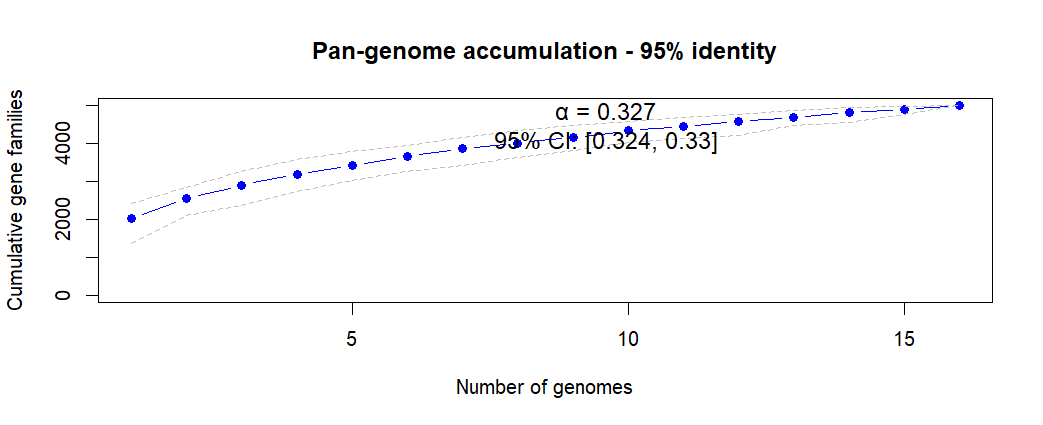


**Supplementary Figure S1** Pan-genome accumulation curve using a 95% identity threshold. The fitted Heaps’ law exponent α = 0.327 (95% CI: 0.324–0.330) remains below 1, demonstrating robustness of the open pan-genome conclusion.


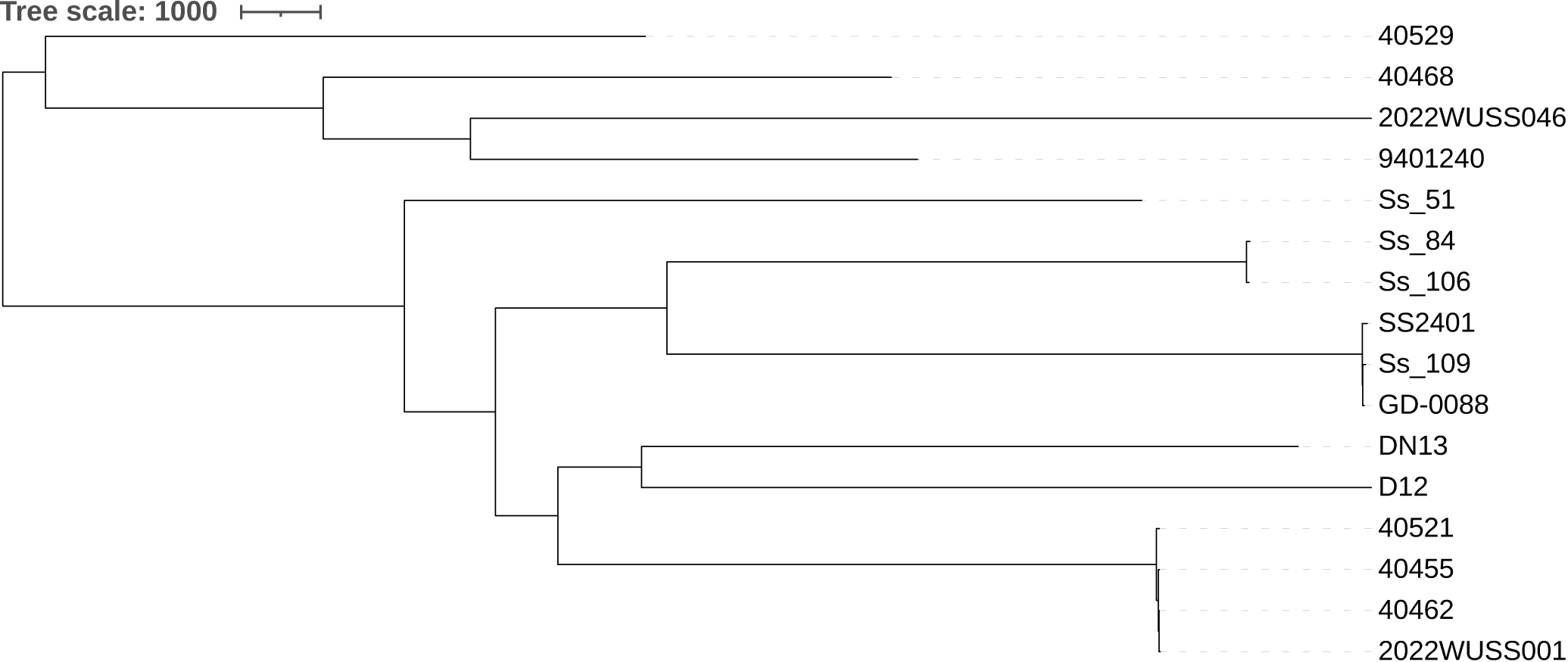


**Supplementary Figure S2** Recombination-corrected phylogenetic tree of *S. suis* serotype 9 strains reconstructed by Gubbins. Branch lengths represent nucleotide substitutions per site. The tree was generated after masking recombinant regions identified in the core genome alignment.

**Supplementary Table S1.** COG functional classification of core and accessory genes in *S. suis* serotype 9.

| **COG category** | **Description** | **Core genes (n)** | **Core genes (%)¹** | **Accessory genes (n)** | **Accessory genes (%)¹** |
| --- | --- | --- | --- | --- | --- |
| J | Translation, ribosomal structure and biogenesis | 133 | 18.9 | 84 | 1.7 |
| K | Transcription | 82 | 11.7 | 336 | 6.7 |
| L | Replication, recombination and repair | 74 | 10.5 | 672 | 13.4 |
| D | Cell cycle control, cell division, chromosome partitioning | 25 | 3.6 | 114 | 2.3 |
| V | Defense mechanisms | 31 | 4.4 | 137 | 2.7 |
| T | Signal transduction mechanisms | 25 | 3.6 | 81 | 1.6 |
| M | Cell wall/membrane/envelope biogenesis | 64 | 9.1 | 155 | 3.1 |
| N | Cell motility | 5 | 0.7 | 24 | 0.5 |
| U | Intracellular trafficking, secretion, and vesicular transport | 18 | 2.6 | 99 | 2 |
| O | Posttranslational modification, protein turnover, chaperones | 37 | 5.3 | 42 | 0.8 |
| C | Energy production and conversion | 39 | 5.5 | 53 | 1.1 |
| G | Carbohydrate transport and metabolism | 105 | 14.9 | 180 | 3.6 |
| E | Amino acid transport and metabolism | 101 | 14.4 | 110 | 2.2 |
| F | Nucleotide transport and metabolism | 59 | 8.4 | 70 | 1.4 |
| H | Coenzyme transport and metabolism | 25 | 3.6 | 72 | 1.4 |
| I | Lipid transport and metabolism | 32 | 4.6 | 28 | 0.6 |
| P | Inorganic ion transport and metabolism | 66 | 9.4 | 81 | 1.6 |
| Q | Secondary metabolites biosynthesis, transport and catabolism | 10 | 1.4 | 35 | 0.7 |
| S | Function unknown | 244 | 34.7 | 874 | 17.5 |
| Unassigned | No COG assignment | 34 | 4.8 | 814 | 16.3 |
| Total |  | 703 | 100 | 5007 | 100 |

¹ Percentages are calculated based on the total number of core genes (703) and the total number of helper genes (5007). Since a gene may belong to multiple COG classes, the sum of the class counts may exceed the total number of genes, so the sum of the percentages may exceed 100%.


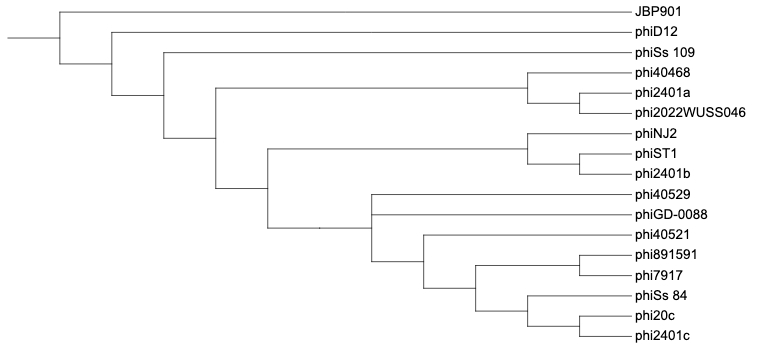


**Supplementary Figure S3** Whole‑genome maximum likelihood tree of the 11 intact prophages. The tree was constructed from a nucleotide alignment of complete prophage sequences using IQ‑TREE with the GTR+F+I+G4 model.

**Supplementary Table S2** Functional modules and virulence genes of the 11 intact prophage

| **Prophage** | **Integrase** | **Terminase** | **Portal** | **Capsid** | **Tail** | **Lysin** | **Sly** | **FeoB** | **Other virulence genes** |
| --- | --- | --- | --- | --- | --- | --- | --- | --- | --- |
| phiGD-0088 | + | + | + | + | + | + | - | - | KsgA |
| phi2401a | + | + | + | + | + | + | + | + | - |
| phi2401b | + | + | + | + | + | + | - | - | - |
| phi2401c | + | + | + | + | + | + | - | - | - |
| phiSs_109 | + | + | + | + | + | + | - | - | SspA |
| phiSs_84 | + | + | + | + | + | + | - | - | - |
| phiD12 | - | + | + | + | + | + | - | - | - |
| phi40468 | + | + | + | + | + | + | - | - | SspA |
| phi40529 | + | + | + | + | + | + | - | - | VraR |
| phi40521 | + | + | + | + | + | + | - | + | - |
| phi2022WUSS046 | - | + | + | + | + | + | - | - | - |

+ indicates presence; – indicates absence.
